# Microfluidic one-step synthesis of a metal−organic framework for osteoarthritis therapeutic microRNAs delivery

**DOI:** 10.3389/fbioe.2023.1239364

**Published:** 2023-07-27

**Authors:** Kaiyuan Yang, Min Ni, Chao Xu, Liangliang Wang, Long Han, Songwei Lv, Wenbo Wu, Dong Zheng

**Affiliations:** ^1^ Department of Orthopedics, The Affiliated Changzhou Second People’s Hospital of Nanjing Medical University, Changzhou, China; ^2^ School of Pharmacy, Changzhou University, Changzhou, China; ^3^ Department of Chemistry, Institute of Molecular Aggregation Science, Tianjin University, Tianjin, China

**Keywords:** microfluidic synthesis, metal-organic framework, miR-200c-3p, delivery, osteoarthritis

## Abstract

As a class of short non-coding ribonucleic acids (RNAs), microRNAs (miRNA) regulate gene expression in human cells and are expected to be nucleic acid drugs to regulate and treat a variety of biological processes and diseases. However, the issues with potential materials toxicity, quantity production, poor cellular uptake, and endosomal entrapment limit their further applications in clinical practice. Herein, ZIF-8, a metal-organic framework with noncytotoxic zinc (II) as the metal coordination center, was selected as miRNA delivery vector was used to prepare miR-200c-3p@ZIF-8 in one step by Y-shape microfluidic chip to achieve intracellular release with low toxicity, batch size, and efficient cellular uptake. The obtained miR-200c-3p@ZIF-8 was identified by TEM, particle size analysis, XRD, XPS, and zeta potential. Compared with the traditional hydrothermal method, the encapsulation efficiency of miR-200c-3p@ZIF-8 prepared by the microfluidic method is higher, and the particle size is more uniform and controllable. The experimental results in cellular level verified that the ZIF-8 vectors with low cytotoxicity and high miRNAs loading efficiency could significantly improve cellular uptake and endosomal escape of miRNAs, providing a robust and general strategy for nucleic acid drug delivery. As a model, the prepared miR-200c-3p@ZIF-8 is confirmed to be effective in osteoarthritis treatment.

## 1 Introduction

MicroRNAs (miRNAs) are a class of short non-coding regulatory ribonucleic acids (RNAs) molecules with 18–24 nucleotides sequences, which are widely involved in post-transcriptional regulation of genes (Bartel, 2004; He and Hannon, 2004; Bartel, 2009). Recently, miRNA has been confirmed to be involved in the maturation of osteoblasts and the osteogenic differentiation of precursor cells-mesenchymal stem cells (Lian et al., 2012; Zhang et al., 2016; Celik et al., 2022; Yang et al., 2022). Research on the miR-200 family has gained popularity in recent years, and miR-200c-3p is one of the most representative variables (Klicka et al., 2022). Studies have shown that the expression level of miR-200c-3p is low in osteoarthritis (OA) samples, but the mechanism of action of miR-200c-3p in OA remains unclear (Proctor and Smith, 2017; Klicka et al., 2022). Studying the role of miR-200c-3p in OA through database prediction revealed many potential targets that may be involved in OA-related pathological processes, including: apoptosis (BCL2, XIAP), upregulation of MMPs (VEGFA), hypertrophy of chondrocytes (FLT1, JAG1, VEGFA), stabilization of ECM (ERRFI1, FN1), inflammatory response (IKBKB, NTRK2, VEGFA), angiogenesis (TIMP2, VEGFA) and balance of extracellular skeleton (TUBB3) etc. (Proctor and Smith, 2017). The database prediction also found that its downstream DNMT3A, DNMT3B, NOTCH1, SP1 and ZEB1 genes may be related to cartilage repair and chondrocyte hypertrophy in OA (Davies et al., 2002). Hence, miRNA is a promising drug candidate for treatment of orthopedic diseases, as it can regulate the expression of related genes from epigenetic, transcriptional, and promote cell self-renewal, proliferation, differentiation, apoptosis, and other cellular activities. However, their further clinical applications are limited by their negative charges, poor stability, and limited cell membranes penetration (Vickers et al., 2011; Chen et al., 2015; O'Neill and Dwyer, 2020). There are dense extracellular matrices and highly negatively charged proteoglycans outside chondrocytes, which make it difficult for osteoarthritis (OA)-related drugs to penetrate cells (Barry et al., 2022). Therefore, increasing the concentration and transfection efficiency of gene drugs, and realizing the high permeability of gene carriers have become research hotspots in the use of miRNA gene therapy for OA (Si et al., 2017; Wang et al., 2019; Zhong et al., 2019).

Viral and non-viral vectors are the two primary categories of gene delivery systems used today (Pouton and Seymour, 2001; Pack et al., 2005; Keles et al., 2016; Boucher et al., 2020; Duan et al., 2021; Sharma et al., 2021). Although viral vectors have high delivery efficiency and transfection efficiency, there are problems such as high manufacturing cost, cumbersome preparation process, low gene load, immunogenic reaction, and carcinogenicity (Kamiya et al., 2001; Capasso et al., 2013; Balakrishnan and David, 2019; Sung and Kim, 2019; Boucher et al., 2020). While non-viral vectors, including dendrimers, liposomes, cationic polymers, and inorganic nanoparticles, *etc*., are capable of overcoming the aforementioned flaws in viral vectors, their efficiencies of distribution and transfection are frequently lower than those of viral vectors (Yin et al., 2014; Foldvari et al., 2016; Miller et al., 2017; Caffery et al., 2019; Yan et al., 2022). Therefore, to develop new non-viral vectors with improved biocompatibility and gene delivery effectiveness is highly desirable to address the above high toxicity and low transfection issues.

Metal-organic frameworks (MOFs) are porous structures composed of metal ions linked by organic ligands, which hold great promise in biomedical applications due to their high surface area, high load capacity, good biocompatibility, and biodegradability (Stock and Biswas, 2012; Furukawa et al., 2013; Wu and Yang, 2017; Li et al., 2019; Feng et al., 2022). With open porous structures and multiple metals or organic active sites, various biomacromolecules (protein and nucleic acid, etc.,) can be integrated with MOFs through three main methods of chemical grafting, physical adsorption, and encapsulation to form MOF-biomacromolecule composites (Abanades Lazaro et al., 2017; Wang et al., 2017; Wang et al., 2018; Yang and Yang, 2020; Lawson et al., 2021). The zeolite-like imidazole skeleton (ZIF) is a subfamily of metal-organic framework (MOF) compounds that contain M-Im-M formed by self-assembly methods (where M stands for zinc and cobalt and Im stands for imidazolate joints) (Sun et al., 2020). The topology of ZIF’s crystal structure is identical to that of aluminosilicate zeolites. In contrast to the tetrahedral silicon or aluminum skeleton of zeolite, which is connected by oxygen atoms, ZIF substitutes a connection of transition metals (Zn or Co) and Im for the tetrahedral silicon or aluminum atoms bridged by oxygen. Since the hybrid framework structure promises greater flexibility in surface modification, ZIFs have advantages over zeolites in applications. So far, ZIF synthesis methods mainly include hydrothermal method (Jian et al., 2015), microwave-assisted method (Tran et al., 2020) etc. However, the above method is difficult to continuous production. Recently, it has been possible to directly and continuously synthesize homogeneous nanomaterial ZIF because microfluidic technology can precisely manipulate the reaction at the micron scale (Balachandran et al., 2021). In addition, it was found that the synthesis of nanoparticles from metal ions (Au, Mg, Ag, Zn ions, etc.,) and organic ligands (amino acids, peptides, proteins, bases, and sugars, etc.,) has excellent potential for use in the treatment of OA and rheumatoid arthritis (Gao et al., 2019; Li et al., 2020; Li et al., 2021; Barjasteh et al., 2022). For instance, nanoclusters synthesized from Au inhibit inflammation-mediated destruction of bone and cartilage (Gao et al., 2019), and MOFs synthesized from Mg can reduce the expression of IL-1β-mediated inflammatory genes in chondrocytes, thereby inhibiting the inflammatory response of subchondral bone (Li et al., 2020).

**SCHEME 1:**
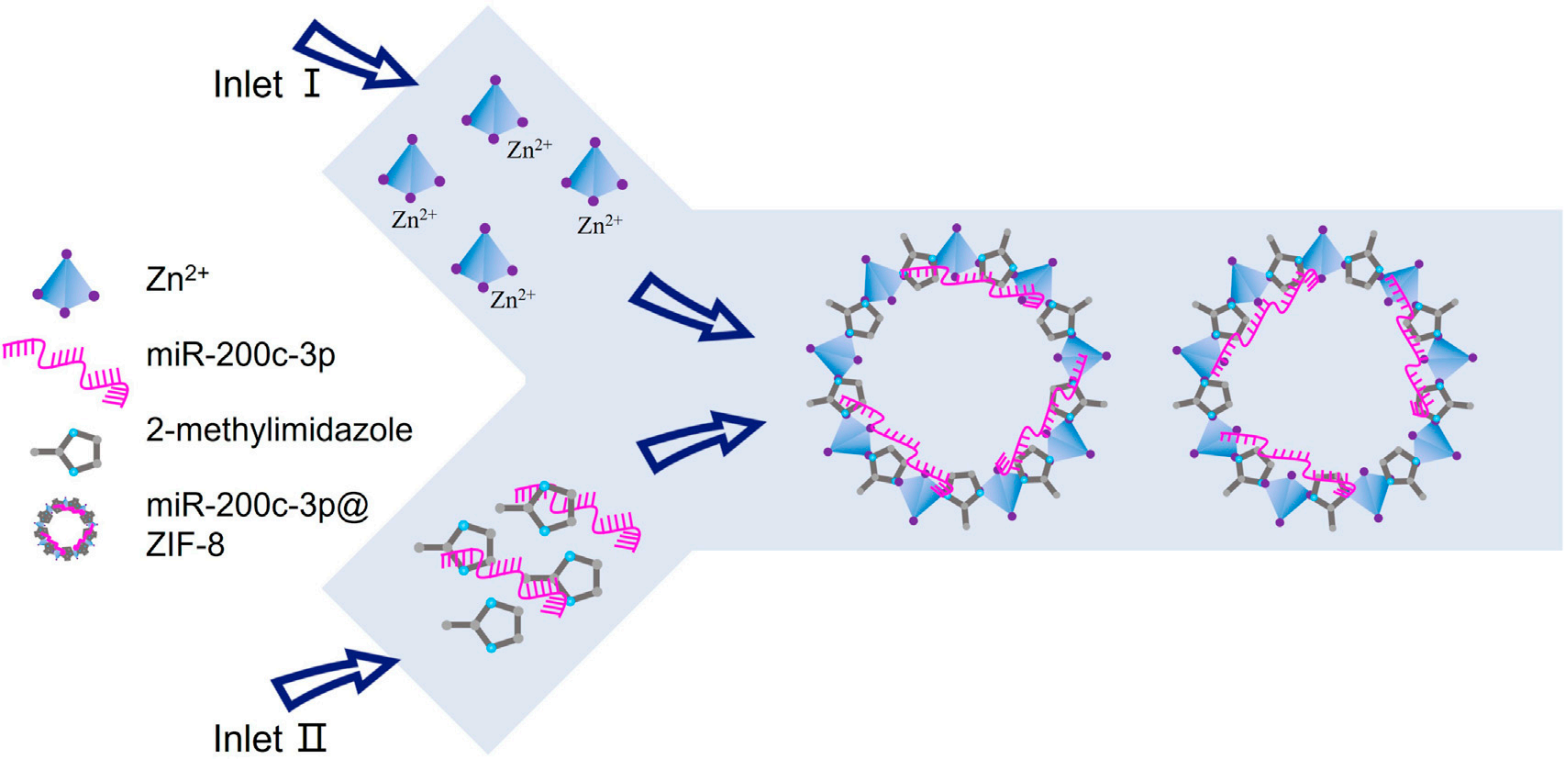
A schematic illustration of the one-step microfluidic fabrication of miR-200c-3p@ZIF-8.

In this contribution, in order to enhance the miRNA transport efficiency, and subsequently regulate miRNA release in response to lysosomal stimulation for improved therapeutic efficacy, ZIF-8, a MOF with noncytotoxic zinc (II) as the metal coordination center, was selected as non-viral vectors to prepare miR-200c-3p@ZIF-8 *via* one single step by a Y-shape microfluidic chip (Scheme 1). As a gene vector, ZIF-8 not only has minimal cytotoxicity and good loading efficiency, but also encourages cellular uptake and improves miRNAs’ capacity for endosomal escape. In addition, since ZIF-8 could release the cargo in acidic endosomes and lysosomes, it is particularly well adapted for intracellular delivery of cargo. Notably, the dissembled product Zn^2+^ ion is an essential component of metalloproteinases, and required for their activity in cartilage (Frangos and Maret, 2020). Further mechanistic research revealed that in lipopolysaccharide (LPS)-induced chondrocytes, miR-200c-3p@ZIF-8 could drastically lower the expression levels of the inflammatory factors, such as IL-1, IL-6, MCP-1, and TNF. This one-step preparation of miRNA vectors by microfluidic chips will provide a robust and convenient tool for gene therapy.

## 2 Materials and methods

### 2.1 Materials

All the reagents were analytical grade and purchased from commercial providers. Lipopolysaccharide (LPS), 5-carboxyfluorescein (FAM) and PCR kit were purchased from Sigma Co., United States. Human chondrocyte CHON-001 and HUVEC (Human umbilical vein endothelial cells) were purchased from ATCC. TRIzol reagent was from Invitrogen. Using a reverse transcription kit, reverse transcription was carried out (TaKaRa Company, Dalian, China). Beyotime Biotechnology Co., was the source of the cell-counting Kit-8 (CCK-8). Luciferase activity was measured with a Luciferase detection kit (Shanghai Yisheng Biotechnology Co., LTD) according to the manufacturer’s protocol. The following primary antibodies were used: GAPDH (mouse mAb, Abcam #ab8245), IL-1 (rabbit mAb, Abcam #abab9722), IL-6 (mouse mAb, Abcam #ab9324), MCP-1 (rabbit mAb, Abcam #ab214819), TNF (rabbit mAb, Abcam #ab183218). Cell lysate aliquots were separated in 10% SDS-PAGE (Beyotime, Code NO. P0012A). Rabbit secondary antibody, mouse secondary antibody, and Lipofectamine^®^ 3000 were purchased from Thermo Fisher Scientific. Deionized water (Millipore Milli-Q Complete) with a resistivity of 18.2 MΩ was used in all experiments. All the solutions were prepared with DEPC-treated water.

### 2.2 Preparation and characterization of miR-200c-3p@ZIF-8 by microfluidics

ZIF-8 nanoparticles were synthesized by a microfluidic swirl mixer chip (Supplementary Figure S1, ESI†). In a typical synthesis, 5.85 mg of Zn(NO_3_)_2_ was dissolved in 4 mL of DEPC-treated water as solution A. 50 μg of miRNA-200c-3p and 40 mL of dimethylimidazole solution (58.90 mg) that had been treated with DEPC were thoroughly combined and agitated for 20 min at room temperature to produce solution B. Then, a syringe pump was used to concurrently inject solutions A and B into the micromixer. Centrifugation (6,000 rpm for 5 min) was used to separate the miR-200c-3p@ZIF-8, which was then cleaned three times to get rid of extraneous reactants. The hydrothermal synthesis of ZIF-8 was based on a previously reported method. Zinc nitrate (20mg/10 mL) was mixed with 2-methylimidazole solution (400mg/10 mL) in a 25 mL bottle of cillin. The mixture was reacted in a vortex for 10 min, placed at room temperature for 30 min, centrifuged (6,000 rpm for 5 min) and washed twice to obtain ZIF-8. The size distribution, zeta potential, and stability of the ZIF-8 and miR-200c-3p@ZIF-8 were evaluated by a nanoparticle analyzer (Nano-ZS, Malvern Instruments Ltd). Transmission electron microscopy was used to analyse the morphology and particle size of ZIF-8 and miR-200c-3p@ZIF-8 (TEM, JEM-2100F). ZIF-8 and miR-200c-3p@ZIF-8 prepared on the microfluidic chip were analyzed using X-ray diffraction (XRD, Rigaku, Smart lab 9). The infrared spectra of ZIF-8 and miR-200c-3p@ZIF-8 prepared on the microfluidic chip were measured by Fourier transform infrared spectrometer (FTIR, Bruker VERTE X70). Cellular uptake efficiency was analyzed by fluorescence imaging of cells using a Nikon C2+ confocal microscope (Nikon, Japan). The transfection efficiency of miRNA-200c-3p@ZIF-8 in CHON-001 cells was quantified by RT-qPCR (ABI7500qPCR).

### 2.3 Encapsulation efficiency and pH-responsive drug release

By using a nanodrop spectrophotometer (Nanodrop Technologies), the encapsulation efficiency (EE) of miRNA-200c-3p in the miRNA-200c-3p@ZIF-8 was measured. The formula used to determine the EE (%) was: EE (%) = 𝑊_𝐸_⁄𝑊_𝑇_ ×100%, where *W*_E_ were the amount of miR200c-3p contained in the miRNA-200c-3p@ZIF-8 and W_T_ was the amount of total miRNA-200c-3p added during preparation. We studied the miRNA-200c-3p release kinetics and degradation of miRNA-200c-3p@ZIF-8 at different pH. A specific concentration of miRNA-200c-3p@ZIF-8 (2 mg/mL) is dispersed in PBS solution with pH ranging from the pH 5.5 to 7.4. The formula used to determine the miRNA-200c-3p release percentages is release percentage (%) = *M*_i_/*M*_t_, where *M*_i_ is the quantity of miRNA-200c-3p which was released and M_t_ is the total amount of miRNA-200c-3p loaded.

### 2.4 Agarose gel electrophoresis

To find out whether miRNA-200c-3p@ZIF-8 may condense, an agarose gel electrophoresis experiment was conducted. The complexes of miRNA-200c-3p and ZIF-8 were electrophoresed at 110 V. The identically produced polyplexes were additionally incubated for 1 h at 37°C in the presence of 10 mM DTT to test the ability of DNA to condense under reducing circumstances.

### 2.5 *In vitro* hemolysis assay

Each subject provided written informed consent, and the Nanjing Medical University Ethics Committee approved all the experimental protocols in accordance with the university’s policies on laboratory and animal care ([2020]KY004-01). Healthy human whole blood from the Affiliated Changzhou Second People’s Hospital of Nanjing Medical University was used in the hemolysis assay. We adhere to the Helsinki Declaration of 1983’s tenets. So, this approach is followed in all of the experiments presented in this study. By centrifuging blood at 3000 rpm for 5 minutes, human red blood cells (hRBCs) were extracted and diluted ten times in PBS. Thereafter, PBS and Triton X-100 dissolved in PBS were used as the corresponding positive and negative controls. Co-incubated with diluted hRBCs for 2 h at 37°C with gentle mixing, test materials made in PBS were then centrifuged at 3000 rpm for 5 minutes. Moreover, 100 μL aliquots of the resultant supernatant were put into a 96-well plate, where hemoglobin release was measured by a microplate reader using the absorbance at 540 nm (Varioskan LUX). The following calculation was used to calculate the percentage of hemolysis:

$$Relative\;rate\;of\;hemolysis\;\left(\%\right)=\left({Abs}_{540\;nm\;with\;miRNA-200c-3p@ZIF-8}-{Abs}_{540\;nm\;in\;PBS}\right)\;/\;\left({Abs}_{540\;nm\;with\;0.1\;\%\;Triton\;X-100}-{Abs}_{540\;nm\;in\;PBS}\right)\times 100\%$$

### 2.6 Cytotoxicity

According to the instructions of Cell Counting Kit-8 (CCK-8, Sigma-Aldrich), the proliferation of CHON-001 and HUVEC cells was detected. For each group, each experiment was run in triplicate. Briefly, 1 × 10^4^ CHON-001 cells and HUVEC cells were seeded in 96-well plates and cultured for 12 h. The cells were then exposed to a succession of groups including blank, ZIF-8, PEI 25 k for 24 h 10 μL of the CCK-8 solution were added to each well, and it was then incubated at 37°C for 2 h. A microplate scanner was used to measure the absorbance of cells at 450 nm of wavelength (Varioskan LUX).

### 2.7 Cellular uptake, and localization of miR-200c-3p@ZIF-8 *in vitro*

To compare the cellular uptake of miRNA-200c-3p@ZIF-8 prepared by microfluidics and Lipo3000, the fluorescence intensity of intracellular 5-carboxyfluorescein (FAM)-miRNA-200c-3p was qualitatively and quantitatively analyzed using confocal laser scanning microscope (C2+,Nikon) and flow cytometer (BD C6 PLUS). At a density of 3 × 10^4^ cells/well in a culture medium, CHON-001 and HUVEC cells were seeded in a confocal plate. Cultured cells were incubated with fluorescently labelled miRNA-200c-3p@ZIF-8 nanoparticles (blank group, miRNA-200c-3p@Lipo3000, miRNA-200c-3p@ZIF-8) in a transfection solution for 24 h before being rinsed with PBS. The cells were then cultured for an additional 24 h after being exchanged for medium that contained 10% FBS. Hoechst 33342 was used to highlight the nuclei for 30 min, after which the cells were examined under CLSM. Using flow cytometry, the cell uptake of fluorescently tagged nanoparticles was evaluated at a minimum of 1 × 10^4^ cells gated per sample. Three duplicates of each trial were carried out.

### 2.8 Internalization pathway of FAM-miR-200c-3p@ZIF-8

CHON-001 cell (1 × 10^4^ cells/well) was treated with FAM-labelled miRNA-200c-3p for 12 h and endolysosome escape between the Liposome 3000 and miR-200c-3p@ZIF-8 was assessed and compared by fluorescence imaging. The cells were then washed twice with PBS before being labelled with Lysotracker (red) to show endo-/lysosomes and Hoechst33342 (blue) for the nuclei. The changes of green and red fluorescence at different transfection times of 3 h and 12 h were detected by CLSM. The Pearson correlation coefficient of green fluorescence and red fluorescence was measured by the Image-processing software (ImageJ).

### 2.9 Chondrocyte culture and *in vitro* model of osteoarthritis

From the American Type Culture Collection, the human chondrocyte cell line (CHON-001) was obtained (ATCC). All cell culture additives and medium were bought from Gibco. The DMEM (Dulbecco’s Modified Eagle Medium) was used to develop the CHON-001 cell. 10% fetal bovine serum (FBS), 4 mM L-glutamine, and 1% penicillin-streptomycin were added to all medium as supplements. Following an incubation with a 0.25% trypsin digestion solution, the cells were allowed to round out and the space between them to widen. At this point, the trypsin was discarded, and the full medium containing serum was added to stop the digestion process and blow the cells into individual ones. Cells were grown at 37°C and 5% CO_2_ (CLM-170B-8-NF, ESCO). CHON-001 cells were seeded in 96-well plates, 5 × 10^4^ cells per well, and stimulated with LPS (10 ng/mL) for 12 h after cultured for 6 h to simulate the OA model *in vitro*.

### 2.10 RT-qCR analysis

With a density of 2×10^5^ cells per well, CHON-001 cells were added to 6-well plates and left to grow overnight. ZIF-8 at a concentration of 50 μg/mL and miR-200c-3p at a concentration of 100 nM were chosen. ZIF-8, PBS, miR-200c-3p, and miR-200c-3p@ZIF-8 were all applied to CHON-001 cells during a 48-h period. The total RNA was then extracted using the Trizol reagent after cells had been washed twice with PBS (Life Technologies). FastKing RT Kit reverse-transcribed the mRNA (With gDNase). With the ABI7500qPCR System and SuperReal PreMix SYBR Green (TIANGEN), quantitative real-time PCR (qRT-PCR) was carried out (Life Technologies). Data were analyzed by using the 2^-△△Ct^ method. The primer sequences used in this study were as follows: upstream primer: AAT​ACT​GCC​GGG​TAA​TGA​TGG​A; downstream primer: CTC​TAC​AGC​TAT​ATT​GCC​AGC​CAC (Table S1).

### 2.11 Western blotting analysis

With a density of 2 × 10^5^ cells per well, CHON-001 cells were planted in 6-well plates and let to incubate for 24 h. ZIF-8 at 50 μg/mL and miR-200c-3p at 100 nM were chosen. ZIF-8, PBS, miR-200c-3p, and miR-200c-3p@ZIF-8 were all applied to CHON-001 cells for 48 h. After post-transfection, CHON 001 cells were washed twice with ice-cold PBS, and Total Extraction Sample Kit was used to capture the proteins from the cells (KeyGEN BioTECH, Nanjing, China). Supernatants were then obtained after being centrifuged for 10 min at 12,000 rpm. The BCA Protein Assay Kit was used to evaluate the protein content of sample proteins (KeyGEN, China). Then, 20 μg of proteins from all the samples were analyzed using SDS-PAGE before being electroblotted onto PVDF membranes from Merck Millipore in the United States. After being blocked for 2 hours with 5% dried milk (made with TBST solution), PVDF membranes were treated with the matching primary antibodies at 4°C overnight, rinsed in TBST, and then incubated for 2 hours with the peroxidase-coupled secondary antibody. An automatic chemiluminescence imaging system was used to detect particular proteins (ImageQuant LAS 4000).

### 2.12 Statistical analysis

Statistical analysis of the data was performed using SPSS 16.0 (SPSS Inc.). All results of at least 3 parallel samples were expressed as mean ± standard deviation (SD). Correlation analysis using Pearson statistical method, statistical significance was present as **p < 0.01; ***p < 0.001.

## 3 Results

### 3.1 Preparation and characterization of miR-200c-3p@ZIF-8 nanoparticles

**FIGURE 1:**
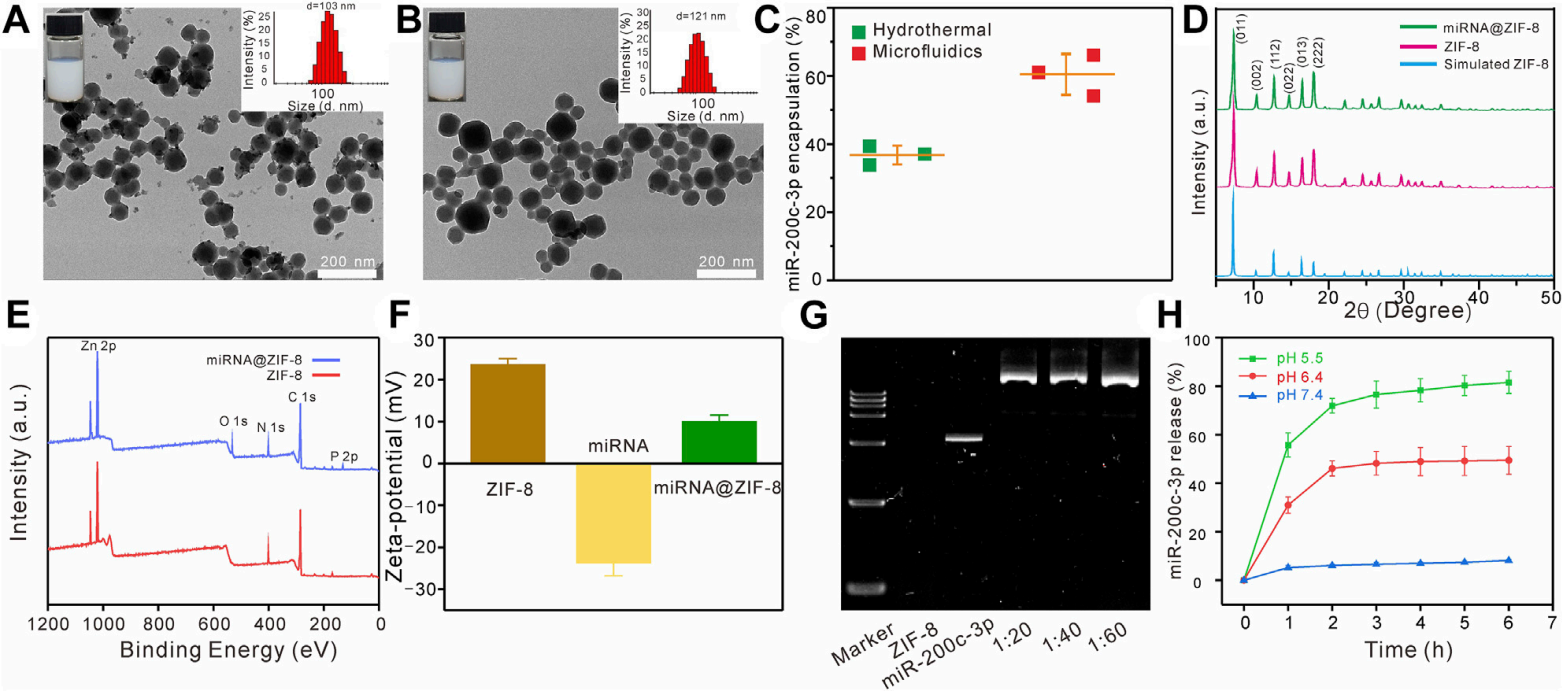
Characteristics of the miR-200c-3p@ZIF-8. **(A–B)** TEM images of ZIF-8 and miR-200c-3p@ZIF-8. **(C)** Compared with the traditional hydrothermal method, the encapsulation efficiency of miR-200c-3p@ZIF-8 prepared using the microfluidic method was higher. **(D)** XRD patterns of miR-200c-3p@ZIF-8, synthesized ZIF-8, and simulated ZIF-8 crystals. **(E)** XPS survey spectra of ZIF-8 and miR-200c-3p@ZIF-8. **(F)** Zeta potential of ZIF-8, miR-200c-3p and miR-200c-3p@ZIF-8. **(G)** Agarose gel shifts of miR-200c-3p@ZIF-8 at different miR-200c-3p/ZIF-8 wt ratios. **(H)** *In vitro* mRNA release profiles of miR-200c-3p@ZIF-8 in phosphate-buffered saline (PBS) buffer with pH 5.5, 6.4, and 7.4.

The TEM images showed that the ZIF-8 had a clear polyhedron form with dimensions of 103 ± 4 nm (Figure 1A), similar to the miR-200c-3p@ZIF-8 with an average size of about 121 nm and a polyhedron form (Figure 1B). After miRNA loading, the polydispersity index (PDI) of miR-200c-3p@ZIF-8 was 0.32, slightly higher than bare ZIF-8 (0.22, Supplementary Figure S2, ESI†). ZIF-8 was prepared using a hydrothermal method as reported elsewhere with modifications. ZIF-8 can be prepared by mixing zinc nitrate solution with dimethylimidazole solution, reacting on a vortex for 10 min and leaving at room temperature for 30 min. UV-vis absorption spectroscopy was employed to measure the quantity of miR-200c-3p in the supernatant before and after encapsulation (Supplementary Figure S3, ESI†), and it was clear that most of the miR-200c-3p has been loaded into the MOFs with an encapsulation rate of 60.35% ± 4.61%, much higher than that of the hydrothermal control (36.67% ± 2.14%, Figure 1C). Change the ratio of miRNA and ZIF-8 to 1:20, 1:40, 1:60, and found that the particle size of the prepared miR-200c-3p@ZIF-8 has little change (Supplementary Figure S4, ESI†). However, different flow rate ratios have a greater impact on the particle size of miR-200c-3p@ZIF-8 (Supplementary Figure S5, ESI†). Fourier transform infrared (FT-IR) results also demonstrated the successful complexation of miR-200c-3p with ZIF-8 (Supplementary Figure S6, ESI†). The powder’s X-ray diffraction (XRD) patterns of both ZIF-8 and miR-200c-3p@ZIF-8 displayed the same distinctive peaks to that of the simulated one (Figure 1D), demonstrating the formation of the intended ZIF-8, and miRNA is loaded by physisorption. ZIF-8 has a large specific surface area of 1,266 m^2^ g^-1^ (Supplementary Figure S7, ESI†), which is beneficial to loading miR-200c-3p. In comparison with the X-ray photoelectron spectrum of ZIF-8, there were two obvious new peaks of O1s (531 eV) and P2p (131 eV) in the spectrum of miR-200c-3p@ZIF-8, confirming the successful loading of miR-200c-3p on ZIF-8 (Figure 1E). After miRNA loading, the Zeta-potential was decreased from 23.6 ± 1.4 to 10.1 ± 1.5 mV (Figure 1F), confirming the adsorption of negatively charged miR-200c-3p on positively charged ZIF-8 again. Additionally, the miR-200c-3p@ZIF-8 was stable as evidenced by the fact that, after 4-weeks storage, neither the particle size nor the PDI substantially increased (Supplementary Figure S8, ESI†). Agarose gel electrophoresis was used to examine the binding preference of miR-200c-3p in ZIF-8, and the results showed that the miR-200c-3p was fully bound when the weight ratio was greater than 1:20 (Lanes 4, 5, and 6 in Figure 1G), indicating that miR-200c-3p was trapped inside ZIF-8 without release.

After incubation at pH = 7.4 for 6 h, less than 10% of miR-200c-3p can be released, while these values were enhanced to 50%, and 82%, at pH = 6.5, and 5.5, respectively (Figure 1H). That is because the coordination bond between zinc and imidazole could be dissociated in an acidic environment, leading to the broken down of ZIF-8, and subsequent miRNA release.

### 3.2 Hemolysis assay and cytotoxicity

**FIGURE 2:**
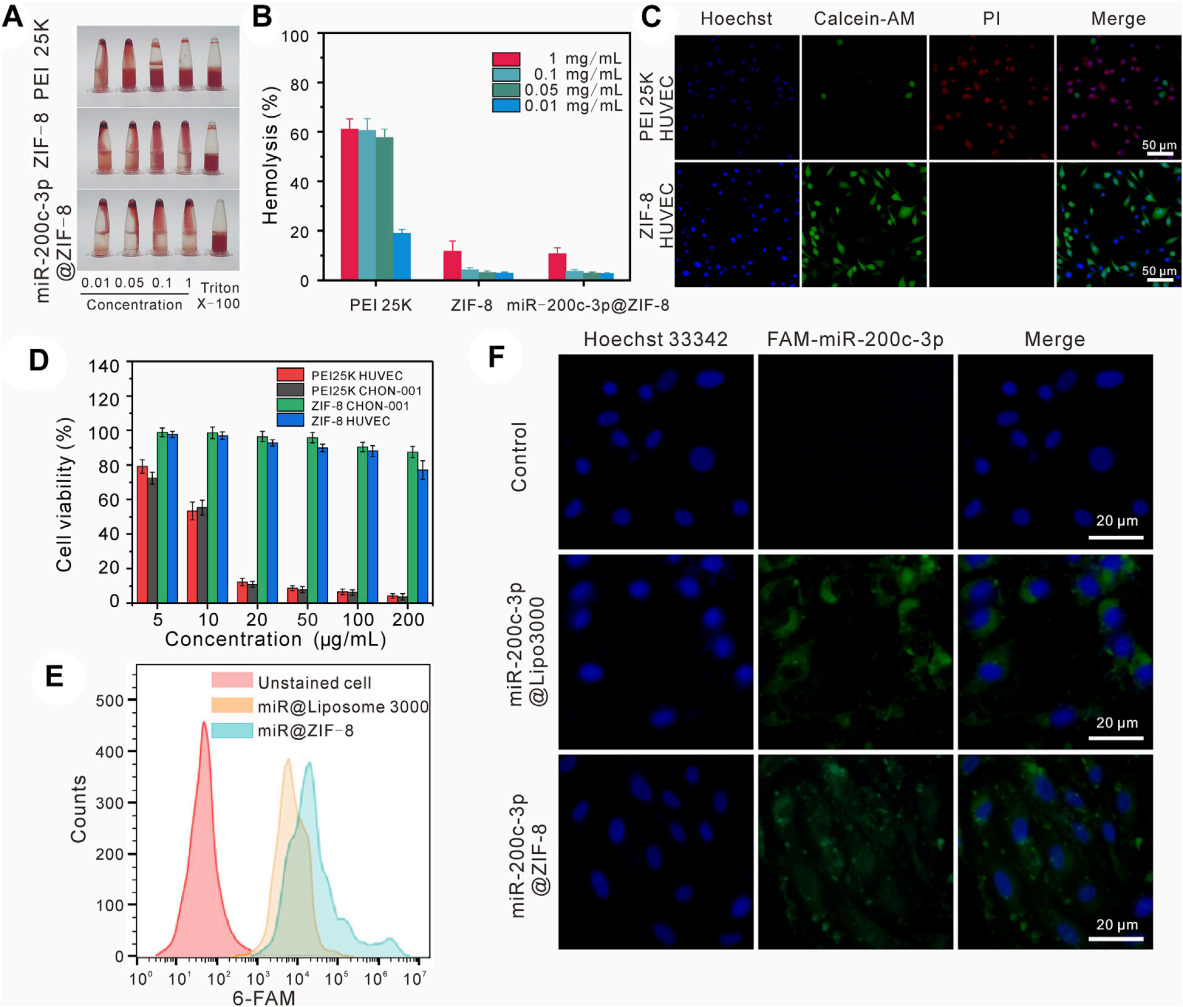
Cytotoxicity and cellular uptake of miR-200c-3p@ZIF-8. **(A–B)** Hemolytic activity of PEI 25K, ZIF-8, miR-200c-3p@ZIF-8. **(C)** Fluorescent images of HUVEC cells incubated with PEI 25 K and ZIF-8, stained with Calcein AM (green) and PI (red), respectively. **(D)** Cytotoxicity assay of ZIF-8 and PEI 25 K at various concentrations for 24 h in different cell lines. **(E)** Flow cytometry analysis of cellular uptake with miR-200c-3p@Lipo3000 and miR-200c-3p@ZIF-8. **(F)** CLSM images of cellular uptake of free miR-200c-3p, FAM-miR-200c-3p@Lipo3000, and FAM-miR-200c-3p@ZIF-8 after 24 h in CHON-001.

As shown in Figures 2A, B, the hemolytic effects of ZIF-8 and miR-200c-3p@ZIF-8 were much lower than the “Gold-standard” transfection agent PEI 25 K. Even at a relatively high concentration of 1 mg/mL, there is no obvious blood cell lysis (less than 20%) in ZIF-8 and miR-200c-3p@ZIF-8 groups. ZIF-8, miR-200c-3p, and miR-200c-3p@ZIF-8 cytotoxicity was examined using the live/dead staining (Figure 2C) and Cell Counting Kit-8 (CCK-8) assay (Figure 2D) on the CHON-001 and HUVEC cell lines, with PEI 25 k serving as the reference. It was clear that at concentrations lower than 100 μg/mL, ZIF-8, miR-200c-3p and miR-200c-3p@ZIF-8 did not impair cell viability and encouraged cell proliferation.

### 3.3 Cellular uptake, and localization of miR-200c-3p@ZIF-8 In vitro

The cell uptake and *in vitro* transfection efficiency of miR-200c-3p@ZIF-8 were quantitatively analyzed by flow cytometry and RT-qCR. The positive control for fluorescence expression was liposome 3000, while the negative control for fluorescence expression was cells that had not been transfected. Compared with the original solution, the fluorescence intensities of Lipo3000 group and miR@ZIF-8 group were only 86.57% and 90.24% of the original intensity, respectively, when CHON-001 cells were transfected with 5-carboxyfluorescein (FAM)-labeled miR-200c-3p for 24 h (Figure 2E). Following that, chondrocyte cultures were introduced to miR-200c-3p@ liposome3000 and miR-200c-3p@ZIF-8. From the confocal images (Figure 2F), it was obvious that the experimental group and the positive control group’s cells displayed a clear bright green emission in contrast to the control group. RT-qCR results showed that compared with the control group, the expression of miR-200c-3p in the transfection group miR-200c-3p@ZIF-8 and Lipo3000 was significantly increased (Supplementary Figure S9, ESI†).

### 3.4 Internalization pathway of FAM-miR-200c-3p@ZIF-8

**FIGURE 3:**
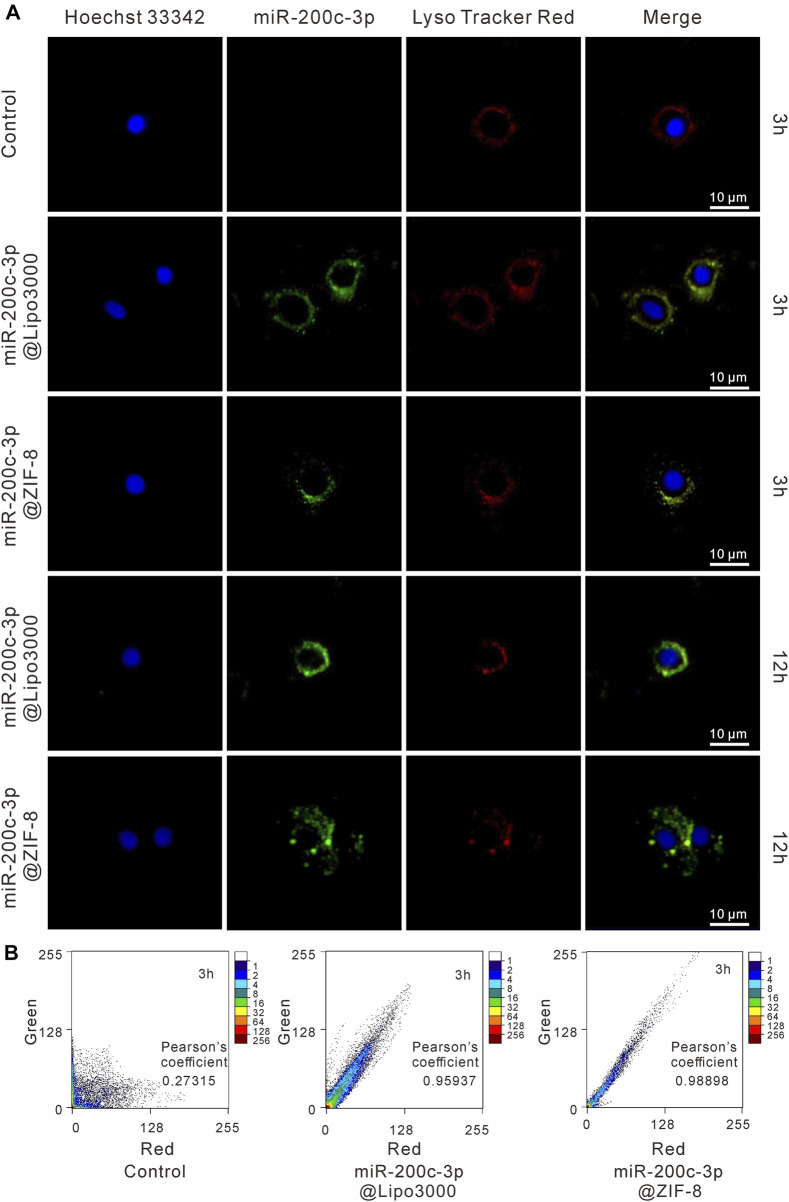
Intracellular localization of miR-200c-3p in CHON-001 cells was detected by CLSM after 3 h and 10 h, respectively. The scale bar represented 10 μm. **(A)** The nucleus was labeled with Hoechst (blue), miR-200c-3p was modified with FITC (green) and Lysosomes were labeled with LysoTracker Red (red). **(B)** Intensity-based colocalization scatterplot and Pearson’s coefficient.

As shown in Figure 3A, the green fluorescence FAM-miR-200c-3p@ZIF-8 was mostly co-located with red fluorescence endosomes/lysosomes after incubation for 3 h, indicating that miR-200c-3p@ZIF-8 was internalized into cells through the endocytic pathway. Using ImageJ software, the fluorescence colocalization between endosome/lysosome (red) and FAM-labeled miRNA (green) was quantified to determine the endosome escape efficiency (Figure 3B), and the Pearson correlation coefficients were calculated as 0.98898 and 0.95937 in miR-200c-3p@ZIF-8 and miR-200c-3p@Lipo3000 groups. When the incubation period was increased to 12 h, the green emission of FAM-labeled miR-200c-3p increased in both groups while the red fluorescence decreased due to the reduction of intracellular lysosomes caused by miR-200c-3p escape (Figure 3A).

### 3.5 The miR-200c-3p@ZIF-8 promotes osteogenic differentiation *in vitro*

**FIGURE 4:**
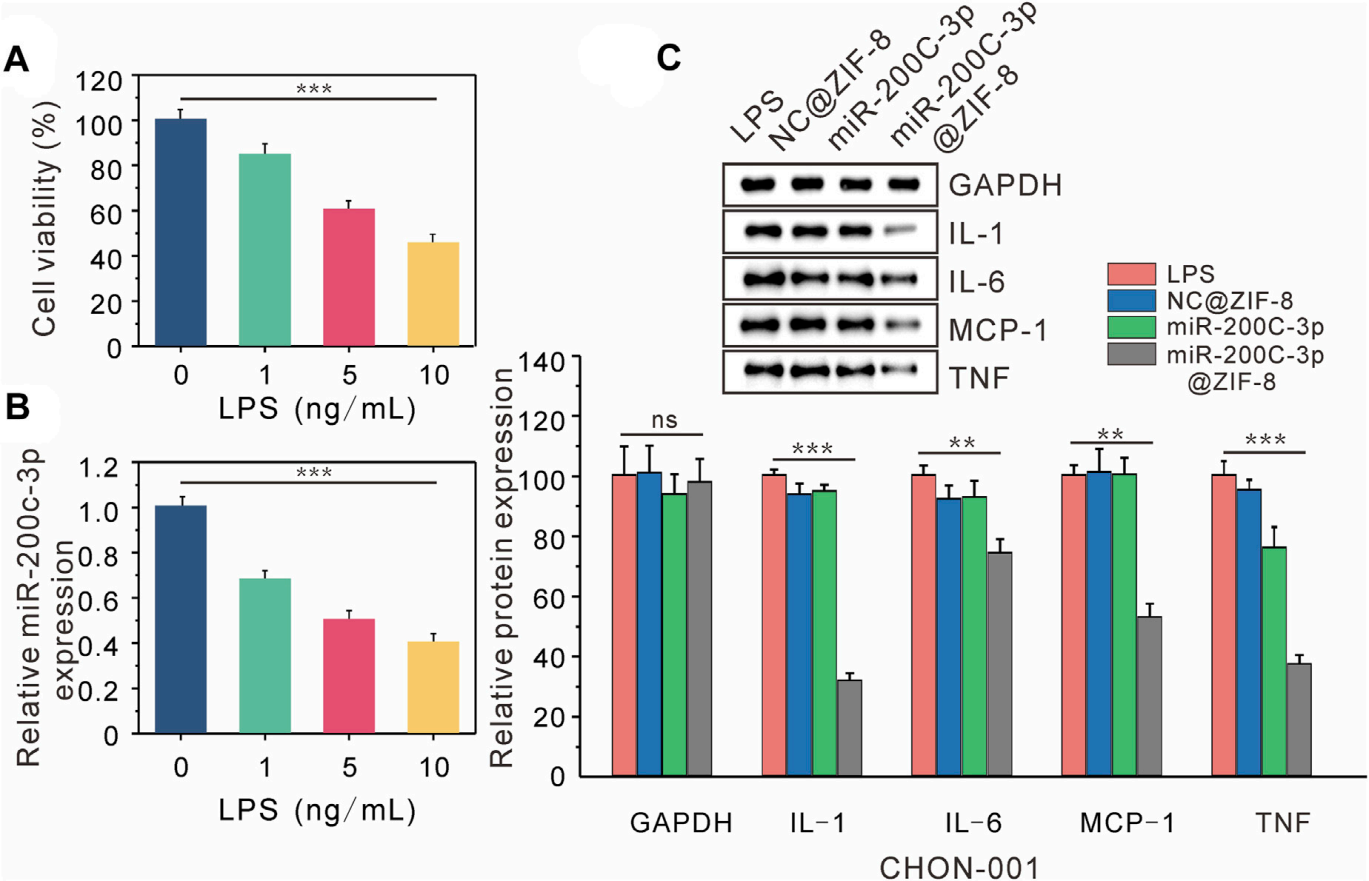
Delivery of miR-200C-3P in LPS-induced chondrocytes modulates inflammatory responses. **(A)** The viability of CHON-001 cells treated with LPS (0, 1, 5, 10 ng/mL) for 12 h was determined by CCK-8. **(B)** The expression of miR-200c-3p in CHON-001 cells treated with LPS (0, 1, 5, 10 ng/mL) was detected by RT-qPCR. **(C)** The effect of LPS, NC@ZIF-8, miR-200c-3p, miR-200c-3p@ZIF-8 on the protein levels of GAPDH, IL-1, IL-6, MCP-1, and TNF.

After CHON-001 cells were exposed to various amounts of LPS (0, 1, 5, 10 ng/mL) for 24 h. According to CCK-8 experiments, cell viability declined as LPS concentration increased, which was decreased to 50% at 10 ng/mL LPS concentration (Figure 4A). In subsequent experiments, CHON cells were treated with 10 ng/mL LPS for 12 h to establish an osteoarthritis model. According to the RT-QPCR data, the expression level of miR-200c-3p decreased steadily with the increase of LPS concentration (Figure 4B), indicating that the expression level of miR-200c-3p was decreased in LPS-stimulated chondrocytes.

The expression of associated proteins was examined by Western blotting, after LPS, LPS + NC@ZIF-8, LPS + miR-200c-3p, and LPS + miR-200c-3p@ZIF-8 treatments were applied to CHON-001 cells. LPS can drive CHON-001 cells to express a lot of inflammatory factors, as seen in Figure 4C The expression of TNF protein was slightly decreased in the miR-200c-3p group, whereas the expression of IL-1, IL-6, MCP-1, and TNF protein was significantly decreased in the miR-200c-3p@ZIF-8 group.

## 4 Discussion

Recent years have seen a rise in interest in the miR-200 family, and miR-200c-3p is one of the most typical variables. Studies have revealed that osteoarthritis samples have low levels of miR-200c-3p expression. As it can regulate the expression of associated genes through epigenetic and transcriptional sources and encourage cell self-renewal, proliferation, differentiation, and death, miRNA is a viable therapeutic option for the treatment of orthopedic diseases. However, its clinical applications are limited by its negative charge, poor stability, and limited cell membrane penetration. Therefore, the development of novel gene drugs to improve the stability and transfection efficiency has become a focus of research in the treatment of orthopedic diseases by miRNA. Given the low toxicity of its constituent parts, electrostatic and coordinative interactions, and large specific surface area, ZIF-8 was selected for nucleic acid drug delivery in this work (Zheng et al., 2016; Li et al., 2019; He et al., 2021). Herein, the preparation of nano ZIF-8 and the miRNA loading were integrated in one single step by Y-shape microfluidic chip (Fig. S1, ESI†) to improve the synthetic efficiency (Zheng et al., 2016; Lv et al., 2021). Specifically, the metal coordination center of Zn^2+^ was injected from the first thoroughfare into the jet mixer, while the ligand and the miRNA (miR-200c-3p in this work) were injected from another entrance, leading to the continuous formation of MOF-miRNA composites, *i.e.* miR-200c-3p@ZIF-8. The results show that compared with the traditional hydrothermal method, the use of microfluidic chips can quickly prepare miR-200c-3p@ZIF-8 with uniform particle size, small dispersion coefficient and high encapsulation rate, and is conducive to industrial expansion production. The miRNA release behaviors of miR-200c-3p@ZIF-8 in buffers with different pH values (pH = 7.4 mimicking normal physiological environment; pH = 6.4 mimicking inflammatory microenvironment; and pH = 5.5 mimicking endosome/lysosome environments) (Luo et al., 2017; Cao et al., 2022; Dong et al., 2022) were subsequently evaluated by nanodrop spectrophotometer, while the standard curve of miR-200c-3p was presented in Fig. S10, ESI†. In pH 5.5 environment, the release rate of miR-200c-3p could reach 82%, indicating that many miR-200c-3p@ZIF-8 could release loaded miR-200c-3p in the lysosome environment.

Generally, the use of nanocarriers in therapeutics is restricted by the interplay between cationic nanocarriers and elements in blood, which eventually results in the reticuloendothelial system clearing out the particles (RES). To evaluate the carriers’ potential toxicity against human cells, the hemolytic activity of the PEI 25K, ZIF-8, and miR-200c-3p@ZIF-8 in fresh human red blood cells (hRBCs) was assessed. From the results, compared with PEI 25K, ZIF-8 and miR-200c-3p@ZIF-8 hardly cause hemolysis. The biological metrics of cell viability and cytotoxicity are crucial for assessing the biocompatibility of gene vectors. ZIF-8, miR-200c-3p, and miR-200c-3p@ZIF-8 did not appear to inhibit cell survival and promoted cell proliferation at doses less than 100 ng/mL. According to the aforementioned results, ZIF-8 is more advantageous as a vector material and has substantially lower toxicity than PEI 25 K.

Next, we examined whether miR-200c-3p@ZIF-8 could enter and express in chondrocytes. In this experiment, miR-200c-3p was labelled with 5-carboxyfluorescein (FAM) with green emission firstly to realize the visualization of miRNA. CLSM was used to qualitatively analyze endocytosis and transfection efficiency. Lipo3000 group and miR@ZIF-8 group showed clear bright green fluorescence compared with the control group, indicating that both can achieve FAM-miR-200c-3p transfection. Flow cytometry data were used to quantitatively analyze the transfection efficiency, and the fluorescence intensities of Lipo3000 group and miR@ZIF-8 group were 86.57% and 90.24%, respectively. The above results show that miR-200c-3p@ZIF-8 is slightly better than Lipo3000 in transfection efficiency.

The endocytic pathway is the primary route used by many non-viral gene delivery vectors, and endosome escape is the key to increasing the transfection efficacy of these vectors. Herein, the miR-200c-3p@ZIF-8 uptake pathway in CHON-001 cells was detailed studied by specifically detecting endosomes and lysosomes using LysoTracker Red. Green fluorescent FAM-miR-200c-3p@ZIF-8 and red fluorescent endosome/lysosome largely coincided after 3 h of incubation, showing that miR-200c-3p@ZIF-8 was internalized into cells via the endocytic route. When incubation time increased to 12h, the green fluorescence of FAM-labeled miR-200c-3p increased and the red fluorescence decreased, which was due to the decrease of intracellular lysosomes due to the escape of miR-200c-3p. These results showed that miR-200c-3p@ZIF-8 could successfully deliver miR-200c-3p into organelles *via* the intracellular route and escape from the intracellular/lysosome to release into the nucleus, which is the key in preventing intracellular degradation of miRNA and successfully inducing gene expression.

IL-1, IL-6, MCP-1, and TNF are well known important inflammatory cytokines, which are highly expressed in chondrocyte OA models. The expression of miR-200c-3p and inflammatory factors (IL-1, IL-6, MCP-1, and TNF) was examined by RT-qPCR and Western blot analysis to investigate the gene-silencing mechanism of miR-200c-3p@ZIF-8. After CHON-001 cells were treated with LPS, LPS + NC@ZIF-8, LPS + miR-200c-3p, and LPS + miR-200c-3p@ZIF-8, Western blotting was used to determine the expression of related proteins. LPS can cause CHON-001 cells to express a variety of inflammatory mediators, however, miR-200c-3p@ZIF-8 dramatically inhibited the protein expression of IL-1, IL-6, MCP-1, and TNF. It was obvious that miR-200c-3p@ZIF-8 effectively increased the phagocytosis and expression level of miR-200c-3p, and the inflammation of CHON-001 cells was treated.

Due to its transparent structure, high specific surface area and porosity, variable pore size, and ease of chemical functionalization, MOF materials, such as ZIF-8, are regarded as a promising class of nano drug carriers. The advantages of MOF over polymer and inorganic carriers include controlled molecular weight and particle size, simple *in vivo* degradation, and significant advantages in clinical transformation. In addition, the metal core composed of MOF materials plays a variety of biological functions in organisms, which is expected to cooperate with drugs to improve the therapeutic effect. There are also some challenges in clinical application: 1. up to now, studies on the drug loading and release kinetics of MOF have been limited; 2 the preclinical evaluation performance of MOFs-based DDS should be optimized through systematic *in vivo* investigations on its stability, degradation mechanism, and adverse effects on healthy organs in order to reach the clinical development stage of MOF nanoparticles.

## 5 Conclusion

In conclusion, miR-200c-3p@ZIF-8 was successfully prepared by using a y-shaped microfluidic chip for LPS-induced chondrocyte inflammation treatment. Compared with the traditional hydrothermal method, miR-200c-3p@ZIF-8 prepared by the microfluidic method showed higher encapsulation efficiency and more uniform and controllable particle size. Moreover, the ZIF-8 material showed lower cytotoxicity and hemolysis performance than the “Gold-standard” transfection agent PEI 25 K. Cell uptake experiments showed that miR-200c-3p@ZIF-8 was comparable to the commercial transfection reagent Lipo3000 in terms of transfection efficiency and cell expression stability. The Western blotting analysis demonstrated that the expression levels of inflammatory factors IL-1, IL-6, MCP-1, and TNF were significantly decreased after transfection of CHON-001 with miR-200c-3p@ZIF-8. All these results indicate that miR-200c-3p@ZIF-8 is promising for the treatment of osteoarthritis. The restrictions of negative charge, poor stability, and restricted cell membrane penetration of miRNA are avoided by employing zif-8 as a representative drug carrier to deliver miRNA, opening up a new avenue for miRNA therapeutic treatment of osteoarthritis. The delivery system based on MOFs prepared by microfluidic technology may also be useful in the delivery of other nucleic acid drugs, including DNA, siRNA, and lncRNA, for tissue regeneration and disease treatment.

## Data Availability

The original contributions presented in the study are included in the article/Supplementary Material, further inquiries can be directed to the corresponding authors.
