# Supplementary material for: Microfluidic one-step synthesis of a metal−organic framework for osteoarthritis therapeutic microRNAs delivery
Source: Front Bioeng Biotechnol. 2023 Jul 27;11:1239364. doi: 10.3389/fbioe.2023.1239364 (PMC10415039; doi:10.3389/fbioe.2023.1239364)
Supplement: Supplementary file 1 [file DataSheet1.docx]

Supplementary Material

Microfluidic one-step synthesis of a metal−organic framework for osteoarthritis therapeutic microRNAs delivery

Kaiyuan Yang^‡1^, Min Ni^‡2^, Chao Xu^1^, Liangliang Wang^1^, Long Han^1^, Songwei Lv^*2^, Wenbo Wu^*3^ and Dong Zheng^*1^

^1^Department of Orthopedics, The Affiliated Changzhou Second People’s Hospital of Nanjing Medical University, Changzhou 213003, China.

^2^School of Pharmacy, Changzhou University, Changzhou 213164, China.

^3^Department of Chemistry, Institute of Molecular Aggregation Science, Tianjin University, Tianjin, 300072, China.

^‡^ These authors contributed equally.

*** Correspondence:** Corresponding Author: E-mail: (Songwei Lv) lvsw@cczu.edu.cn; (Wenbo Wu) [wuwb@tju.edu.cn](mailto:wuwb@tju.edu.cn); (Dong Zheng) [2402501054@qq.com](mailto:2402501054@qq.com);

**Table S1** Primer sequence

|  | upstream primer | downstream primer |
| --- | --- | --- |
| miR-200c-3p | AATACTGCCGGGTAATGATGGA | CTCTACAGCTATATTGCCAGCCAC |
| U6 | CTCGCTTCGGCAGCACA | AACGCTTCACGAATTTGCGT |


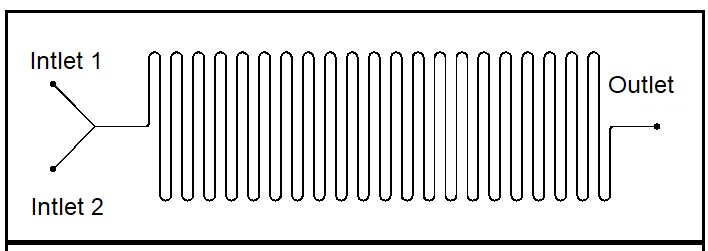


**Fig. S1** Schematic diagram of chip structure used for microfluidic synthesis.


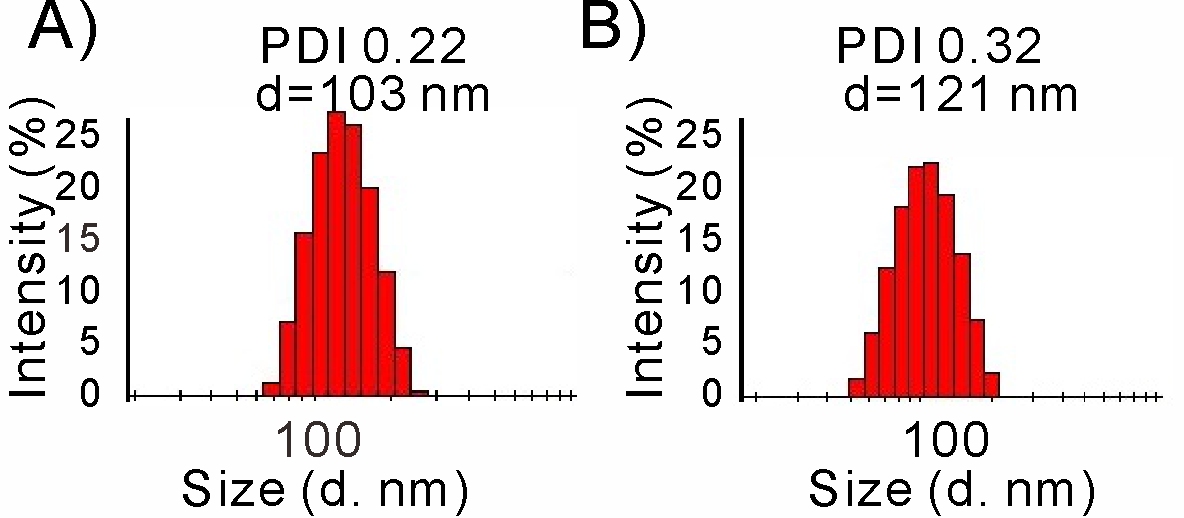


**Fig. S2** Size distribution and PDI of A) ZIF-8 and B) miR-200c-3p@ZIF-8.


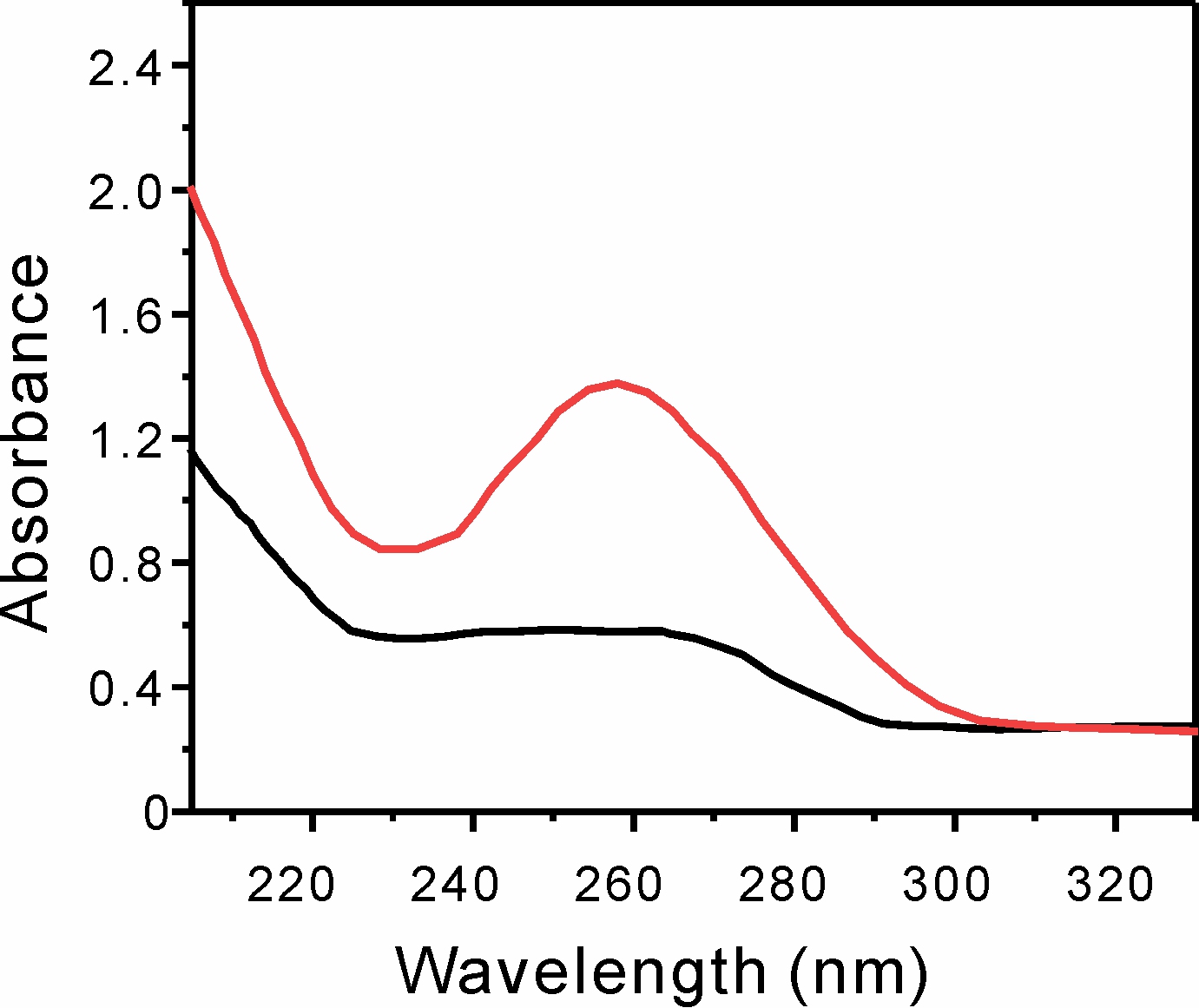


**Fig. S3** The UV–vis spectra of the original solution of miR-200c-3p before encapsulation (red) and the residual miR-200c-3p in supernatant after encapsulated by ZIF-8 (black).


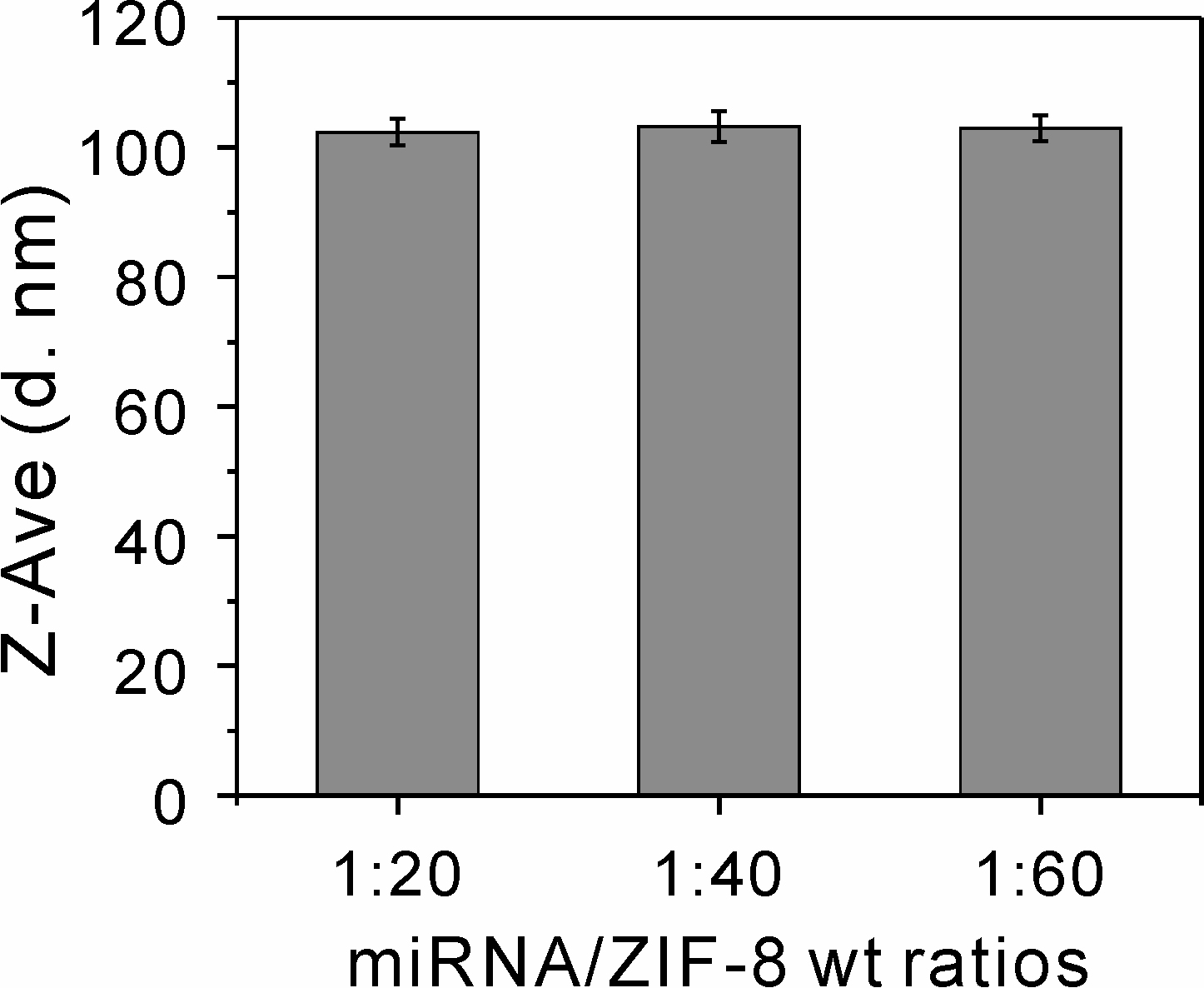


**Fig. S4** The effect of different mass ratios of miRNA and ZIF-8 on the particle size of miRNA@ZIF-8.


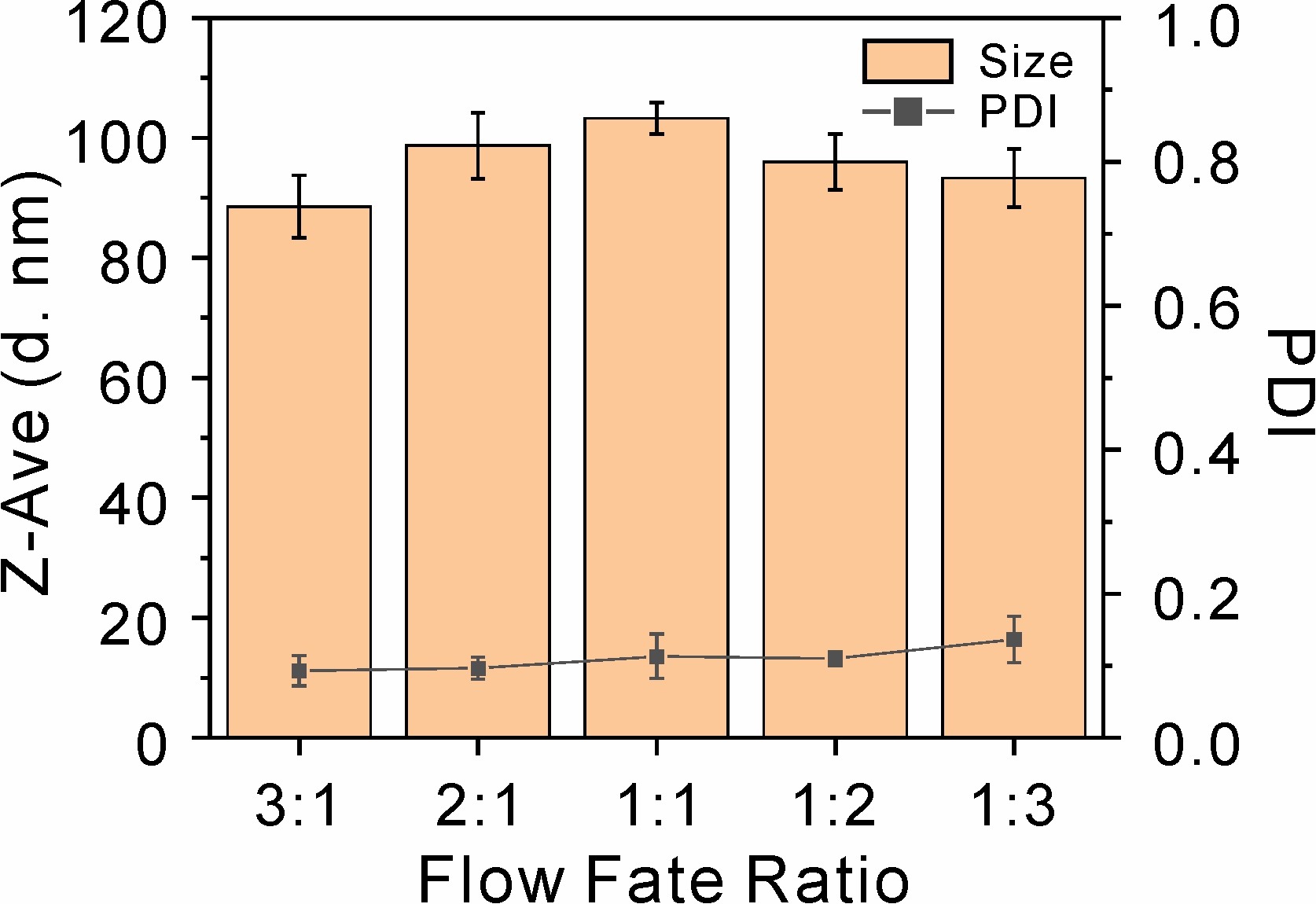


**Fig. S5** The effects of different flow rate ratios (Zinc nitrate solution: miRNA and dimethylimidazole solution) on the formation of miR-200c-3p@ZIF-8.


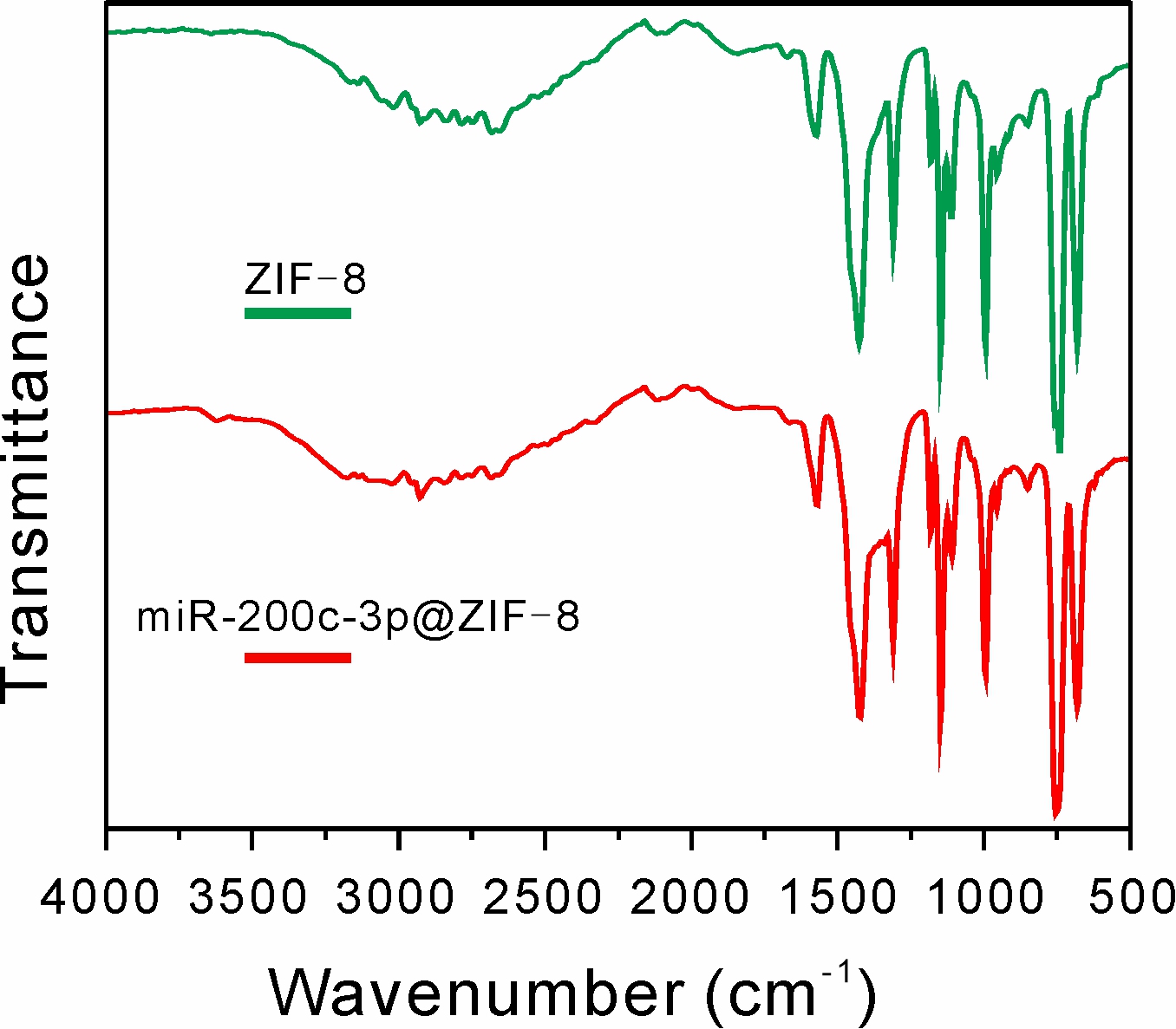


**Fig. S6** Fourier transform infrared spectra of ZIF-8 (green) and miR-200c-3p (red).


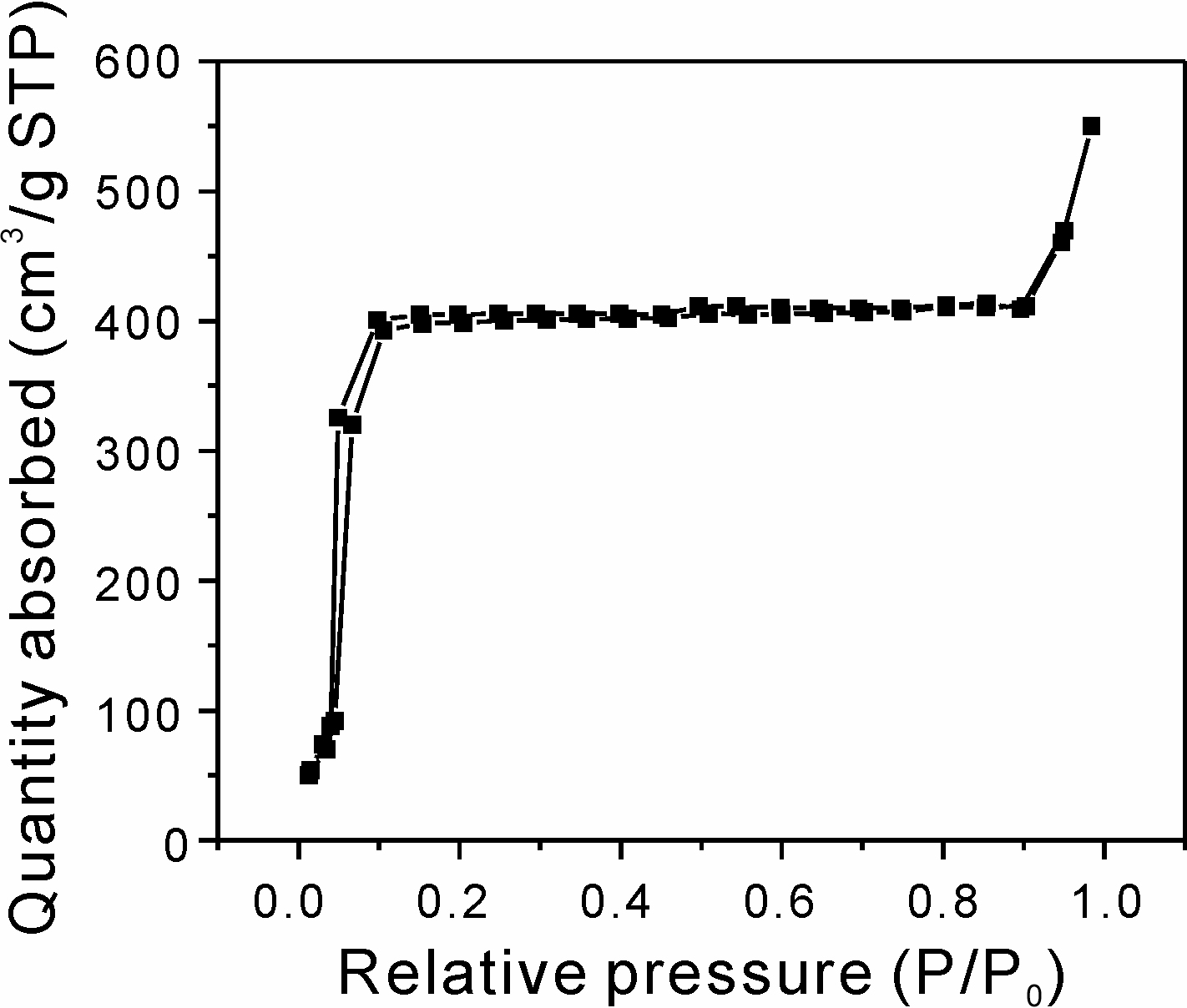


**Fig. S7** N2 absorption-desorption isotherms (h) of the prepared ZIF-8.


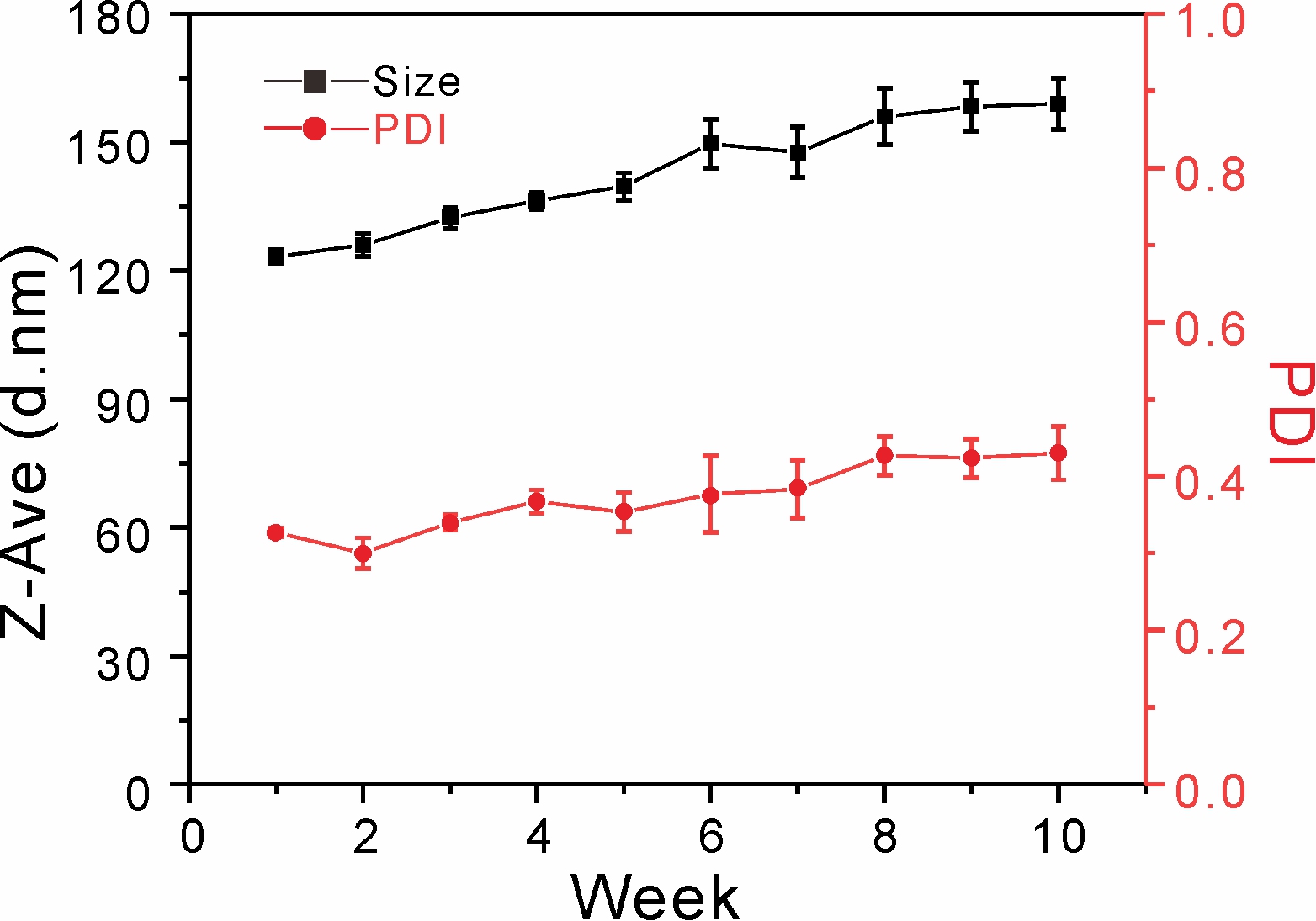


**Fig. S8** Stability of miR-200c-3p@ZIF-8 (4 ℃).


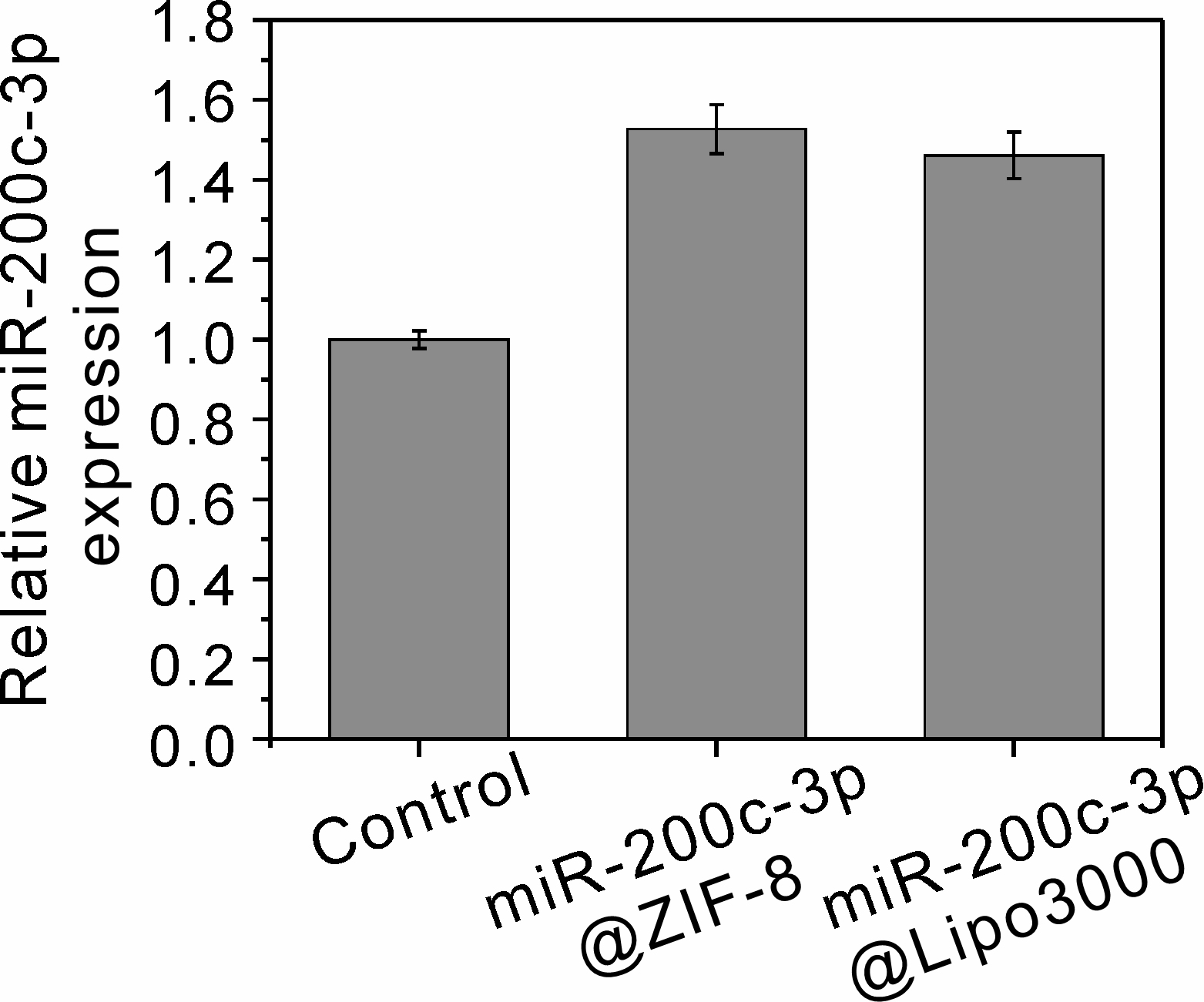


**Fig. S9** The expression of miR-200c-3p in CHON-001 cells treated with miR-200c-3p@ZIF-8 and miR-200c-3p@Lipo3000 was detected by RT-qPCR.


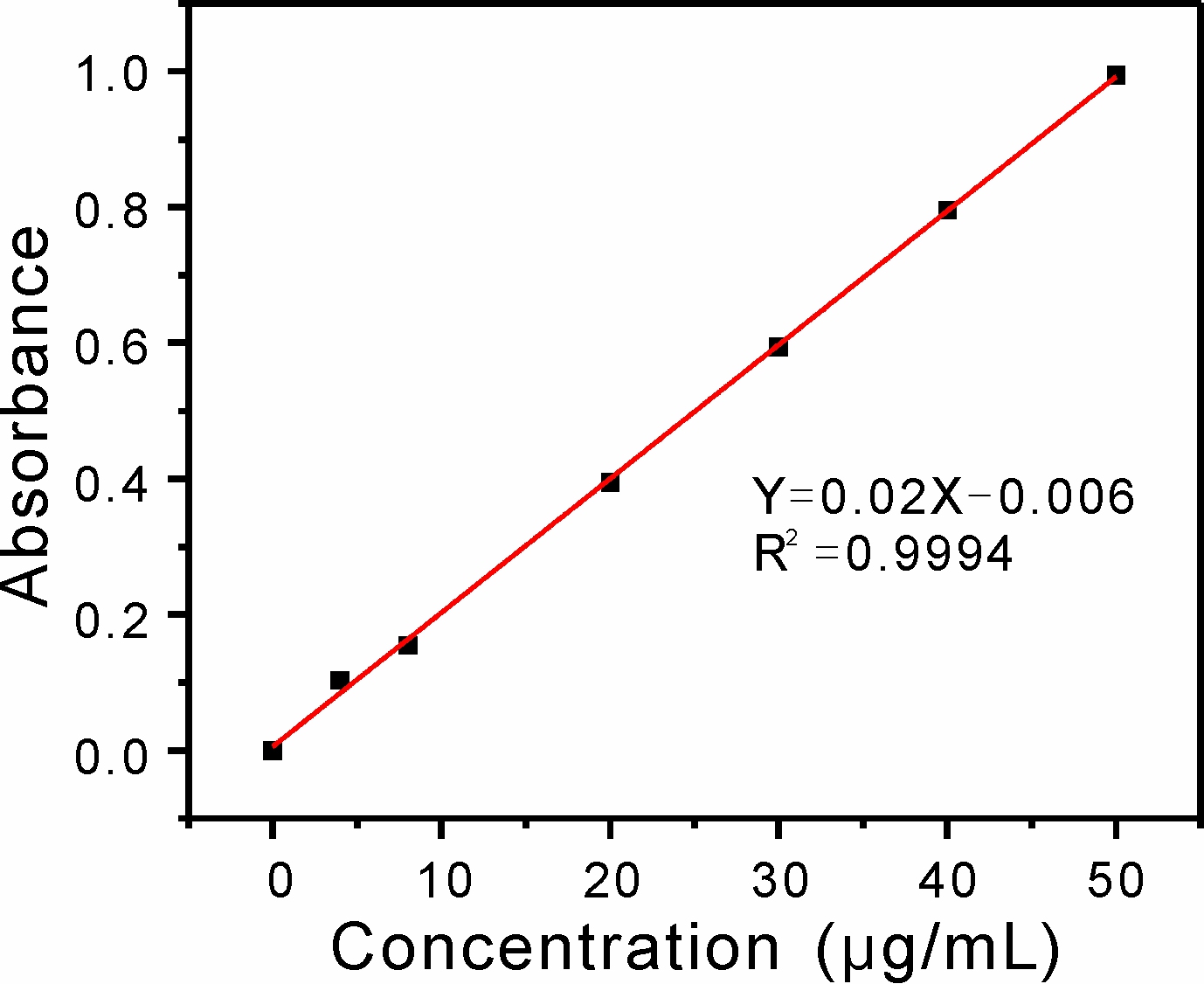


**Fig. S10** The standard curve of miR-200c-3p.

**
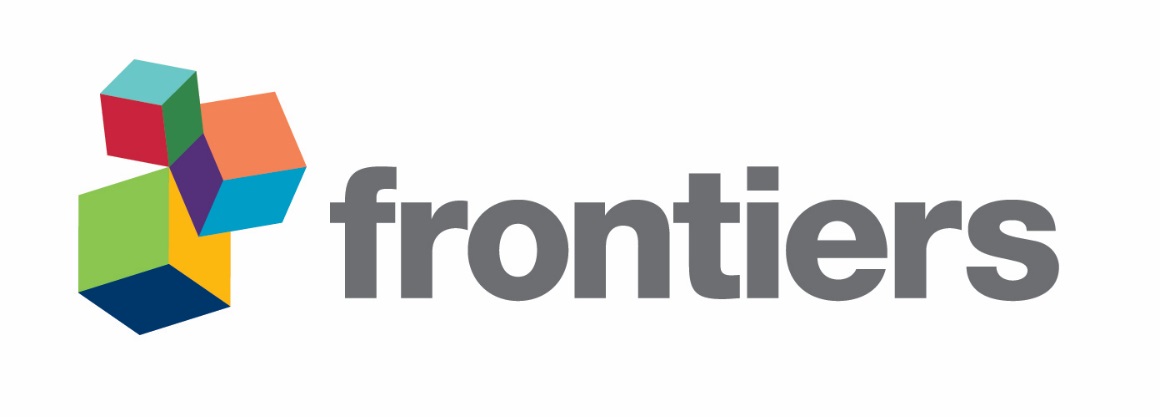
**
