# Supplementary material for: Microfluidic one-step synthesis of a metal−organic framework for osteoarthritis therapeutic microRNAs delivery
Source: Front Bioeng Biotechnol. 2023 Jul 27;11:1239364. doi: 10.3389/fbioe.2023.1239364 (PMC10415039; doi:10.3389/fbioe.2023.1239364)
Supplement: Supplementary file 2 [file Image1.pdf]

Microfluidic one-step synthesis of a metal–organic framework for osteoarthritis therapeutic microRNAs delivery

Kaiyuan Yang, Min Ni, Chao Xu, Liangliang Wang, Long Han, Songwei Lv, Wenbo Wu and Dong Zheng

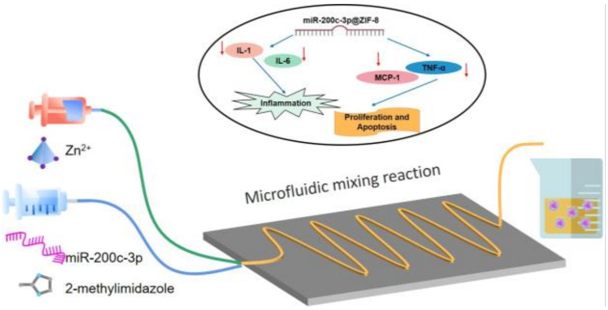

ZIF-8, a metal-organic framework (MOF) with noncytotoxic zinc (II) as the metal coordination center, was selected as miRNA delivery vector to prepare miR-200c-3p@ZIF-8 in one single step by Y-shape microfluidic chip to achieve highly efficient intracellular miRNA release for osteoarthritis therapy.
